# Brewing and the Chemical Composition of Amine-Containing Compounds in Beer: A Review

**DOI:** 10.3390/foods11030257

**Published:** 2022-01-19

**Authors:** Hayden Koller, Lewis B. Perkins

**Affiliations:** School of Food and Agriculture, University of Maine, 5736 Rogers Hall, Orono, ME 04259, USA; hayden.koller@maine.edu

**Keywords:** beer, brewing, biogenic amine, amino acid, yeast, *Saccharomyces*, *Brettanomyces*, lactic acid bacteria, adjunct

## Abstract

As microbreweries have flourished and craft beer brewing has expanded into a multibillion-dollar industry, the ingredients and techniques used to brew beer have changed and diversified. New brewing ingredients and techniques have led to increased concern over biogenic amines in the final product. Biogenic amine composition and concentration in beer, as well as the changes to the protein and amino acid content when adjuncts are used, have received little attention. A complex biochemical mixture, the proteins, amino acids, and biogenic amines undergo a variety of enzymatic and non-enzymatic catabolic, proteolytic, and oxidative reactions during brewing. As biogenic amines in fermented food receive increased scrutiny, evaluating knowledge gaps in the evolution of these compounds in the beer brewing process is critical.

## 1. Introduction

Beer brewing has been practiced for thousands of years by many cultures and continues to be a popular and financially significant practice [1]. Unlike fruits and honey, which have free fermentable sugars, barley and other cereal grains used for beer require a combination of malting and mashing to metabolize their complex starches into maltose and other simple sugars before fermentation can be initiated [2]. Malting is a complex biochemical process during which barley grains are allowed to germinate and produce a variety of carbohydrase and proteolytic enzymes and other proteins necessary for the brewing process [3]. During mashing, the malt is steeped in water and heated to between 60 and 70 °C to activate the carbohydrase enzymes, with the goal of metabolizing the complex starches into simple mono and disaccharides usable for fermentation [4]. This resulting sweet liquid, with high concentrations (around 45% by weight) of maltose, is called wort. The saccharides in wort are subsequently converted to alcohol and various flavor compounds by yeast fermentation, but the fate of the residual enzymes, free amino acids, and proteins are also important.

The proteins and amino acids present in beer serve a multitude of functions. Proteins affect many properties of a beer, including the mouthfeel, color, flavor, and stability. These compounds are also catabolized by yeast for energy, reproduction, and enzyme generation [5]. Proteins and the amino acids that compose them vary widely in foods. Identification of these nitrogenous compounds present in barley and wort, and which are most likely to be metabolized by *Saccharomyces* yeast, is necessary for predicting the amino acid and biogenic amine composition of the finished beer.

Biogenic amines are a diverse group of basic, nitrogenous, amine-containing molecules. Amines in this group are primarily created through the enzymatic decarboxylation of amino acids by living organisms, but can also be generated through transamination of various alkanes, such as the conversion of putrescine to spermidine [6]. The presence of these compounds in food products is a concern because of their potential deleterious health effects when ingested. Biogenic amines are potent signaling molecules within the human body; symptoms of biogenic amine consumption can include hypertension, hypotension, migraines, pseudo-anaphylaxis, and gastrointestinal distress [7].

As the beer industry has evolved and microbreweries have become more popular, the characteristics of beer have also changed. What was once a market primarily filled with yellow corn and rice-based lagers is now home to a diverse family of beer styles prepared using a variety of different styles, grains, and adjuncts. While biogenic amines have been well studied in food products like fish and meat, beer is just beginning to be scrutinized. This review examines published literature to highlight gaps in knowledge regarding the catabolic changes proteins experience during beer production, biogenic amine genesis by fermentative microbes, and the effects novel ingredients and brewing techniques have on microbial stress.

## 2. Storage Proteins of Barley

To fully understand the amine profile of beer, the origin of amine compounds in beer must be examined. Most of the proteins, peptides, and amino acids found in beer originate from compounds present in barley (*Hordeum vulgare*). While beer can be fermented from a variety of cereal grains, by far the most common and well-studied is barley. Barley kernels contain a variety of compounds, including lipids, carbohydrates, vitamins, minerals, and proteins, that all influence the sensory properties of a beer [8].

Storage proteins in barley can be divided into four general categories based on their solubility and amino acid profile. Globulins are the fraction of barley proteins soluble in salt solutions [8]. Glutelins are alkali-soluble proteins that, much like globulins, are insoluble in pure water but soluble in various other solutions, including those with high pH. Albumins are water-soluble proteins easily dissolved in water [8]. The final category of proteins are prolamins, specifically, the family of prolamins known as hordeins. Hordeins are alcohol-soluble storage proteins and constitute roughly 50% of the protein content of barley [8]. Due largely to their abundance and the sensory effects and chemical properties their catalytic products impart to beer, hordeins are the most critical of these proteins in brewing.

### 2.1. Globulins

Globulins comprise approximately 15% of all barley proteins and are storage and structural proteins found in the aleurone layer of the barley kernel [8]. These proteins, their role in beer fermentation, the effect they have on the sensory properties, and the chemical makeup of beer is not well understood and require further study.

### 2.2. Albumins

Albumins make up only 3% of all barley proteins and are found in the embryo of the kernel [8]. These proteins play an important role in finished beers’ flavor, mouthfeel, and foam formation. Of the proteins found in barley, albumins are most likely to survive the brewing process and are often found in finished beer due to their heat stability, water solubility, and resistance to heat coagulation [4]. It is believed that albumins are responsible for the foaming ability of beer. These albumins are chemically changed in a variety of ways during the brewing process to convert them into “foaming” proteins. During brewing, albumins are glycated and acylated using free sugars and acyl groups produced during the catalysis of sugars and other compounds [4]. As the brewing process continues, disulfide groups are broken and these modified albumins are unfolded, creating foaming protein.

### 2.3. Glutelins

Glutelins encompass 45% (30 to 50%) of all barley protein and are found in the endosperm of the kernel [8]. These proteins are the major components of gluten, an important structural and functional protein in many foods, and a known allergen [9]. Due to their poor solubility in wort, glutelins are not believed to play a major role in the chemical makeup of beer. The concentration of glutelin in finished beer is high enough to cause an allergic reaction in gluten-sensitive individuals, implying that some glutelins escape the brewing process unchanged [10].

### 2.4. Hordeins

Hordeins make up roughly 45% (30 to 50%) of all barley protein [8]. Hordeins are storage proteins found only in barley, but similar analogues exist in other cereals, including gliadin in wheat and secalin in rye [9]. Hordeins are divided into different groups depending on the size and amino acid profile of the specific hordein molecule. A-hordeins are relatively small, lightweight proteins roughly 15 to 25 kDa in mass [10]. Due to their size and structure, these proteins are unlikely to be categorized as true storage proteins within the barley seed. B-hordeins are medium-sized proteins, 40 kDa in mass, and represent more than 70% of the hordein content of the barley kernel [11]. These proteins, also known as “sulfur-rich hordeins”, have a high content of amino acids that contain sulfur, especially cysteine [10]. Sulfur-rich amino acids allow a high level of cross-linking within these proteins and help to stabilize dough and bread products made from barley. C-hordeins, which are larger than A- and B-hordeins at roughly 60 kDa in mass [11], are also known as “sulfur-poor hordeins” because they lack the high concentration of cysteine found in B-hordeins. Additionally, the low concentration of sulfur-containing amino acids causes these proteins to have less cross-linking within their tertiary structure [10]. D-hordeins are high molecular weight proteins, usually more than 100 kDa [11], that are believed to play a major structural role within the barley kernel due to their size and a high number of cysteine residues [11]. In general, D-hordeins have been less well studied than the other barley proteins.

### 2.5. Amino Acid Profiles of Barley Protein

The amino acids that comprise the hordeins in barley determine which amino acids and biogenic amines may be present in a finished beer. Because barley is a plant, the specific amino acid concentration can vary widely depending on the growing conditions of the crop, such as soil nutrition, climate zone, and rainfall [2]. Moreover, different barley cultivars may produce grains that differ in amino acid content; for example, high-lysine barley is a widely grown cultivar but is rarely used in brewing. The amino acid profile of barley, like most cereal grains, is comprised primarily of glutamic acid and proline [12]. These two amino acids make up nearly 40% of all the amino acids, by dry weight, in the barley kernel (Table 1 and Table 2), with leucine usually the 3rd most abundant (4.5%) and lysine (0.8%), methionine (0.75%), and tryptophan (0.7%) usually the least abundant [2]. Although B-Hordeins are rich in sulfur-containing cysteine, the overall concentration of cysteine is low, comprising about 2.1% of the total amino acid content [2]. With this knowledge, a profile of the potential amino acid/amine content in a final beer product can be constructed, as barley is the predominant source of these nitrogenous compounds.

## 3. Effects of Malting and Mashing on Proteins and Amino Acids

Malting, kilning, and mashing are three interconnected brewing concepts necessary for beer production. During the malting process, barley grains are allowed to germinate, leading to the creation of a variety of carbohydrase and protease enzymes [2]. After germination, the seeds are dried in specialized kilns to drive off moisture and develop various flavor compounds. Lightly kilned grain retains high levels of several different enzymes, while highly roasted grains lose enzymes to thermal denaturation but develop dark colors and stronger flavors as well as various roasted flavors through the Maillard reaction [13]. During the Maillard reaction, reducing sugars, such as glucose and maltose, react with free amino acids to create a variety of brown pigments, as seen in foods such as bread, and a variety of desirable flavor compounds in the finished beer [14]. Kilning causes a variety of changes in the protein makeup of the grains due to pyrolysis, caramelization, Maillard reactions, and denaturation, among other reactions, but the specific changes that occur are not well known and require further study. Mashing is the process of steeping malt in hot water to gelatinize and convert long-chain starches from the malted grain into fermentable sugars. The malt and water mixture is then heated to specific temperatures, 60 and 70 °C, which promote the activity of various carbohydrase enzymes that cleave amylase and other starches into fermentable sugars, mainly glucose and maltose [15]. The concentrated sugar and water solution produced by mashing is known as wort and is analogous to must in wine production.

### 3.1. Malting and Malt

During malting, several key steps occur that have lasting impacts on the chemical composition and sensory characteristics of the completed beer. One of the most critical steps is the creation of various carbohydrase enzymes such as alpha-amylase and beta-amylase within the seed kernel. These enzymes are crucial to the brewing process as they allow the unfermentable barley starches to be catalyzed into simple sugars. However, the formation of these carbohydrases is only one of many processes that occur during malting. Proteolytic enzymes that are produced within the seed kernel contribute to the germination process, as they are necessary to metabolize the various hordein proteins present in barley. Within the barley seed, granules of starch are surrounded by a net-like structure of hordein protein, which forms a water-tight barrier around the carbohydrate [13]. When the germination process begins, the protease enzymes produced by the barley begin cleaving the hordein coat into a variety of smaller water-soluble peptides [16]. Although this process is not well understood, inhibition of hordein catalysis renders the resultant malt almost completely unusable; the starch molecules appear unable to gelatinize as their protective coating is not degraded [13]. While it is known that this process increases the number of water-soluble peptides by removing hydrophobic residues, the specific peptides produced are not well known [17]. The amount of water-soluble protein available in a grain is called the “total soluble protein” and is commonly determined utilizing spectrophotometry [18]. During wort production, the amount of soluble protein increases through the actions of enzymes and the brewing process itself. The increase in soluble proteins is highest during the malting process due to the high enzyme activity during this stage (Table 3), generating water-soluble peptides and proteins. Solubilized protein concentration drops during the wort production (mashing) due to thermal denaturation of coagulation of some of the proteins [18]. Studies have shown that even after this catalytic process, only about 20% of all barley proteins are soluble in the wort [13].

### 3.2. Amino Acid Profiles of Barley Wort

The barley malt is converted into wort via the mashing process. During mashing, the proteolytic and carbohydrase enzymes continue to cleave proteins into soluble peptides and amino acids, and complex starches into simple tri/di/monosaccharides, respectively [3]. Table 3 shows that, in most cases, about 20% of all soluble protein/amino acids are produced during the malting stage [18]. As the wort is boiled, protein-related chemical reactions and processes occur. The high temperature and acidic nature of the wort causes about 70% of all barley proteins to coagulate and precipitate [18]. This first protein removal stage, known as a hot break or “protein crash” [18], removes substances that cause haziness, cloudiness, and poor mouthfeel and produces a clearer beer. The goal of this “protein crash” is not the removal of all protein, only insoluble or coagulated proteins that may impart unpleasant flavors to the final beverage.

All fermentations require free amino nitrogen (FAN) for yeast to reproduce and generate enzymes required for fermentation and survival. Additionally, the soluble proteins provide a variety of sensory characteristics in beer, including mouthfeel and foam retention. Table 4 shows the most abundant amino acids are proline and glutamic acid, while histidine and tryptophan remain at a low overall concentration [19]. This is a profile of amino acids in just these varieties of wort and is not applicable to every beer style, as recipes and heating profiles vary. The grain bill of beer varieties can vary and can include a variety of barley malts (each roasted or treated in different ways or for different lengths of time) and cereal grains (corn, rice, rye, wheat, oats, etc.), which can change the amino acid composition. Additionally, other adjuncts such as fruit, nuts, meat products, etc., commonly added to beer can further modify the chemical composition of a beer.

## 4. Yeast and Fermentation

After wort is created and cooled, yeast is mixed or “pitched” into the wort to ferment the available sugars into ethanol and other byproducts. The yeasts most commonly used for alcoholic fermentation are members of the *Saccharomyces* genus, with the most common of which is *Saccharomyces cerevisiae*. It is well known that in an anaerobic environment, yeast will consume simple sugars and convert them into ethanol, carbon dioxide, and a variety of fusel alcohols and esters. Less widely recognized, is that yeasts also metabolize nitrogenous compounds, especially amino acids, to reproduce and create more catalytic enzymes. This protein digestion contributes to the chemical composition of the final beer as it creates not only biogenic amines but various other nitrogenous compounds as well.

### 4.1. Free Amino Nitrogen

Free amino nitrogen or “FAN” is a critical component of all alcoholic fermentations. FAN is a measurement of the number of available amino acids and digestible peptides in a wort or must. Without a diverse and abundant source of nitrogen, yeast are unable to successfully reproduce and ferment [5]. The degree to which yeast can utilize various amino acids contributes to the final composition of the beer. To digest amino acids yeasts typically produce decarboxylase enzymes which convert amino acids into biogenic amines. There are four categories of amino acids; Group A through Group D, which are categorized by how easily and quickly yeasts can utilize the amino acid (Table 5). Understanding which amino acids are easily digested during the fermentation process provides a more accurate estimate of the final nitrogenous profile of a beer [20]. For example, as glutamic acid is both abundant and easily digested by yeast, a finished beer is expected to have a high concentration of gamma-aminobutyric acid (GABA), the decarboxylated form of glutamic acid. This phenomenon has been observed in other alcoholic beverages, namely grape wine, in which nearly all glutamic acid is converted into GABA [21].

With this information, an amino acid profile can potentially be constructed for a beer, even if the grain bill is highly varied. Because Group A and Group B amino acids are easily digested and absorbed by the yeast (Table 5), there should be few of these amino acids in the finished beer, but their decarboxylated biogenic amines may be present in higher concentrations. In contrast, Group C amino acids typically survive the brewing process intact, so the final beer has lower concentrations of biogenic amines from these amino acids. Group D consists of only proline, the most abundant amino acid in wort, which is almost completely unusable to yeast during fermentation. Although yeast does have enzymes capable of metabolizing proline, such as proline oxidase, these enzymes require oxygen to function [22].

The anaerobic environment that forms during fermentation inhibits the activity of proline-metabolizing enzymes, causing proline to remain mostly unmetabolized [22], though there are exceptions to this process. First, if a wort is highly oxygenated during the lag phase of fermentation, enough oxygen can persist in the wort to allow yeast to digest some proline [23], although this has a minimal effect on the overall proline concentration. A second exception, more applicable to the future, is the potential use of genetically modified yeast. While not yet commercially available, a growing number of yeast strains that can assimilate proline in low oxygen environments have been created by researchers [24]. These yeast strains were developed through the use of genetic modification, specifically utilizing ethyl methanesulfonate random mutagenesis on *Saccharomyces cerevisiae* yeast. In the future, it is likely that these yeast species will see wide use as they allow the utilization of the native nitrogen provided by the barley, negating the need to fortify the wort [24]. Several studies have shown that the order in which yeasts digest amino acids is independent of the amino acid’s concentration [25]. Even with an abundance of valine and a minimum of glutamic acid, yeast cells will consume glutamic acid before the valine. This preference is coded by a variety of genes in the yeast’s DNA and is regulated by several enzymes and the presence of various nitrogen compounds [26].

### 4.2. Yeast-Born Amino Acids

In addition to metabolizing amino acids, yeast are capable of producing amino acids from various precursor chemicals. While yeast are able to produce a variety of amino acids through transamination, this usually only occurs in media with extremely limited concentrations of amino acids; the amino acids that trigger this when they are not present are valine, isoleucine, phenylalanine, glycine, tyrosine, leucine, histidine, lysine, and arginine [5]. While the production of non-proteinogenic amino acids is not well understood, studies have proven that yeast can produce some of these compounds in beer [27]. One such amino acid that can be produced is ornithine, an intermediary in the production of other amino acids [28]. Ornithine can also be transformed into another non-coded amino acid, citrulline. Citrulline is easily converted to arginine, a coded amino acid but also exists in a free form in solutions [29]. While the full breadth of non-proteinogenic amino acids produced by yeasts is not well known, these compounds exist in beer and affect analysis and beer quality, causing unpleasant flavors to form [5]. These amino acids are not usually produced in high concentrations [27] but are often present in beer and detected during chemical analysis.

### 4.3. Autolysis

Finally, yeasts can interact with the amino acid content in beer in one other way; autolysis. As yeasts age and the alcohol content of the beer increases, yeast cells begin going through the process of autolysis. Autolysis occurs when enzymes within the yeast begin catabolizing the yeast cell, causing the cells to lyse and their contents to be released. This process is different from normal yeast death as the catabolic cascade causes a variety of proteins to be digested and converted to free amino acids [30]. Autolysis is generally avoided in the brewing process as it is a source of off-flavors. This usually occurs only in beer that has not been properly aged and is allowed to sit on dead yeast cells, also known as lees, for extended periods of time [31]. As autolysis occurs, the amount of free amino acids increases significantly, while protein-bound amino acids decrease. In some trials, the free amino acid concentration post autolysis increased as much as six-fold [31]. Controlling autolysis during the brewing process is necessary as the released amino acids are both a sensory defect and a spoilage vector. Due to usable carbon scarcity, lactic acid bacteria normally can not survive in the fully fermented beer, but can grow if that beer had autolyzed yeast proteins in it [30]. The amino acid profile varies between different yeast genera and even between strains of the same species depending on the environment they are grown in. An amino acid profile for autolyzed samples of *S. cerevisiae* and *S. stipitis* yeasts (Table 6) shows the variation in amino acid concentration of all amino acids [32]. These samples were prepared by inducing autolysis in a cultured yeast sample using saponins instead of metabolic stress, like in an actual beer. The autolyzing process appears to cause an increase in the levels of certain amino acids such as serine, arginine, and asparagine. When analyzing beer that has been left on its yeast for an extended time, autolysis will influence the nitrogen, amino acid, and biogenic amine profile.

### 4.4. Brettanomyces Yeast

While Saccharomyces yeasts are the primary alcohol producer in most beer, the *Brettanomyces* or “Brett” genus has regained some of its historical popularity. This genus of “wild” yeast found throughout the world behaves differently from traditional brewer’s yeast. While Saccharomyces yeasts have been cultivated for many years to produce alcohol and minimal amounts of other flavor components, Brett yeast is used for the opposite reason. *Brettanomyces* yeast can produce alcohol, but are well known for their ability to produce a cornucopia of pleasant and unpleasant flavor compounds. Producing flavors that range from “fruity” and “spicy” to “horse blanket” and “urine”, these yeasts are equal parts pest and gift [33]. In some beer styles, like sours and lambics, these yeasts are responsible for the many unique flavors associated with those beverages, but in many others, they are considered a flaw or a contaminant [34]. The other unique aspect of these fungi is their ability to survive and thrive in inhospitable environments that many other species of yeasts cannot. Brett yeasts are able to grow in biologically stressful alcohol-rich and acidic environments, such as beer and wine, and can metabolize carbon sources for energy that other yeast species can not, such as lactose, dextrins, and acetic acid [35].

#### 4.4.1. *Brettanomyces* Amino Acid Uptake

Of the many strains of *Brettanomyces* yeasts used in beer brewing, *Brettanomyces bruxellensis* is the most commonly utilized and the most commonly studied. *B. bruxellensis* is unique in that it is able to grow easily in beer (including as a brewing spoilage organism), a food product that is acidic, alcoholic, and lacking in metabolically-usable carbon sources [35]. This yeast genus is able to absorb and metabolize amino acids in a similar fashion to Saccharomyces yeast (Table 7) [36]. Similarities exist between *Brettanomyces* amino acid uptake and Saccharomyces amino acid uptake, with glutamate, glutamine, and aspartate being the preferred amino acid sources (Table 7). They differ slightly in the secondary and tertiary tiers, primarily with serine and alanine being more easily metabolized by Brett and hydrophobic amino acids being more readily consumed by Saccharomyces. This study was done on only a single strain of a single species of Brett yeast, and results should not automatically be considered generalizable to all *Brettanomyces* yeast, as assimilation by Brett yeast can vary greatly from strain to strain [36].

#### 4.4.2. *Brettanomyces*, Proline, and GABA

Two other major amine compounds found in Brett-fermented beer are proline and gamma-amino butyric acid (GABA). Proline, as previously discussed, provides an abundant source of nitrogen in beer and wine, but is mostly unusable to Saccharomyces. Most microbes use an oxygen-based catalytic enzyme to digest proline, but the fermentation of beer occurs primarily in an anaerobic state. There has been some disagreement on whether Brett yeast can utilize proline in a wort/finished beer. A study by Crauwels et al. [37] implies that proline can be used as both a carbon and a nitrogen source by *B. bruxellensis* in wine. This information is mostly refuted by other studies, including work by Blomqvist et al. [38], which showed proline digestion only if oxygen was present. It is safe to assume that proline is only metabolized in beer that has a high enough concentration of oxygen to allow the oxygenase enzyme to function. GABA is an amine compound formed through the decarboxylation of glutamic acid and is formed in large concentrations during fermentation. GABA is also released in large volumes during the autolysis process [32]. Brett yeasts are generally able to digest this amine, especially when it is the only/most abundant source of nitrogen available. When consumed, GABA is converted into glutamate and succinate, which are both further converted into other compounds [35]. The level of metabolism varies greatly from strain to strain, with some *Brettanomyces* strains able to consume 60% of the available GABA, while other strains increase the amount of GABA present by creating more.

In an experiment conducted by Smith et al. [39], the variable nature of GABA consumption was highlighted with 50% of tested strains of *bruxellensis* consuming it, and the rest producing more of the amine. In all species, proline was initially produced in small amounts but went on to be consumed in large quantities, counter-intuitive to the assumption that proline catalysis only occurs in the presence of oxygen. One potential explanation for this phenomenon is that Brett yeasts have an alternative catalysis pathway that is not currently known [39]. The other likely explanation is that proline is not being catabolized but absorbed to repair cellular damage, something commonly seen with microbes exposed to nutrient-poor and high-ethanol environments [35]. With so much unknown regarding Brett yeasts and their catabolic pathways, further research needs to be done.

## 5. Beer

Creating a generalized view of the amino acids, biogenic amines, and proteins present in beer is a considerable challenge. According to the Brewers Association, in 2019, there were well over 120 different recognized styles of beer [40]. Each of these beer styles has its own unique grain bill, aging regimen, fermentation style, adjuncts, microbiota, among other differences, which influence the chemical composition of the final product. The styles represented on this list include unfiltered and highly hopped New England IPAs, high gravity Russian imperial stouts, rye beers, long-aged sour beers, and many more. The effects that these recipes have on a beer’s chemical composition are not well studied, but the published literature provides a glimpse of some potential patterns.

### 5.1. Amino Acid Profile vs. Beer Style

A study done on identifying amino acids in five beer styles is shown in Table 8. In this work, a variety of beer styles were tagged with an amine-specific tagging chemical and separated and analyzed using high performance liquid chromatography with mass spectrometer detection (HPLC-MS) [41]. The three types of alcoholic beer and two types of non-alcoholic beer analyzed in this study were stylistically similar. Pils and Altbier are both types of lagers, while wheat beer is brewed with significant quantities of wheat; all three are “simple” beers without novel adjuncts or long aging methods. The data shows that both broad similarities and stark differences exist between these simple styles. Some amine-containing compounds like proline and ethanolamine were consistent amongst all five beer styles, but the majority of analyzed compounds were found at inconsistent levels. This variation highlights the potential influences of beer diversity, as even with three simple, similar beer styles, stark differences exist.

While it makes sense that different beer styles have diverse amine profiles, studies have shown variation exists even within a single style of beer. An evaluation of a variety of lager beers from the Czech Republic is shown in Table 9. This study revealed that within this style of beer, amino acid profiles are relatively consistent [42]. Researchers determined that concentrations of amino acids may vary in beer from a single country, but claim that this is largely due to differences in wort concentration used to produce the beer [42]. Beer samples from different countries, even if controlled for the concentration of wort, still show variation in amino acid concentration. This variation in concentration is normal for plant-derived products because the climate that a grain is grown in, can have a drastic effect on the nutritional and biochemical profile of grain.

These results are consistent with other limited studies focused on the amino acid content of beer. Within a given style, different brands of beer are likely to have similar amino acid profiles, but variation can occur when considering the cultivar, growing location, and agricultural practices such as fertilization rate and irrigation utilized when growing the grain. Between different styles of beer, there can be significant differences in amine content due to the myriad of different ingredients and techniques utilized.

### 5.2. Albumins, Haze Proteins, and Foaming Proteins

As previously discussed, some proteins manage to escape the heating, separating, and fermenting steps and persist in the final beer. These proteins are critical for the finished beer’s flavor, mouthfeel, and foam formation. The barley proteins most likely to survive in a final product are heat-stable albumins, as they readily dissolve in water and resist coagulation [16]. These albumins are chemically changed in a variety of ways during the brewing process and are converted into “foaming” proteins. During the brewing process, albumins are glycated and acylated using free sugars and acyl groups produced during the catalysis of sugars and other compounds [4]. As the brewing process continues, disulfide bonds are broken and these modified albumins are unfolded, creating foaming proteins [4]. The other major source of proteins found in beer are the haze-forming proteins. These substances are most commonly formed from the previously discussed hordeins, some of which avoid complete catalysis by enzymes and heat coagulation and are soluble in alcohol-rich beer [43]. These proteins become less soluble and form hazes when they bind to the various polyphenols present in beer. When analyzing nitrogen-containing compounds in beer, neither haze nor foaming proteins are likely to cause separation problems during HPLC analysis due to the large disparity in size between the comparatively small dipeptides, amino acids, and biogenic amines, and the high molecular weight proteins, ~300 Da vs. 30,000 Da.

## 6. Biogenic Amines

Another class of nitrogenous compounds to consider in beer are biogenic amines. Biogenic amines are a group of amine-containing nitrogenous bases formed by the decarboxylation of amino acids or transamination of other compounds and are formed by many living organisms. Biogenic amines are a family of diverse, powerful signaling molecules and include such chemicals as histamine, serotonin, tyramine, and cadaverine, among many others. These compounds are of interest to scientists as they have deleterious effects when consumed in food products. When consumed biogenic amines can cause a plethora of side effects, including migraines, high/low blood pressure, pseudo anaphylaxis, and gastroenteritis [44]. There is little comprehensive work describing biogenic amine levels in commercial beer products, especially looking at the effects that unique adjuncts, exotic yeast and bacteria species, high alcohol contents, long aging periods, and other novel techniques have on the final product.

### Biogenic Amines in Beer

Researchers evaluating several Chinese beer varieties found large variations in the content of the amines, tryptamine, phenethylamine, tyramine, putrescine, spermidine, and spermine [45]. These biogenic amines were selected as they are the most common biogenic amines found in fermented foods. The results of this study (Table 10) show a great degree of variation amongst the tested amines. The concentration of some amines, such as histamine, varied 480% between the highest and lowest samples [45]. The levels of amines present in these beer samples vary considerably. Examining beer sample 5, analysis shows that this beverage had a histamine content of 4.62 µg/mL, which is equivalent to 1.64 mg of histamine per 355 mL can of beer. This concentration, equivalent to 4.62 ppm, is far below the FDA’s legal limit of 50 ppm for histamine in fish [46], but this does not mean the product is completely safe. Concerns about ethanol lowering the intoxicating-threshold for biogenic amines could make this concentration dangerous in certain individuals [47]. Studies have shown that histamine-intolerant people experienced biogenic amine poisoning when served a histamine-spiked (4 mg) sample of wine, which would be matched after just 2.5 cans of this beer [7]. While the actual concentration of the biogenic amines was low, the level of variation between beer samples is surprising. All the beers tested were major brands, including Heineken, Corona, and Yanjing, made with similar ingredients, so one would expect the concentrations of biogenic amines to be more consistent. The level of processing, simplicity of ingredients, and quick throughput times for these brews would likely be the cause of the overall low concentration, <10 ppm vs. >200 ppm, of these compounds.

While biogenic amines and their formation in beer are currently not well-understood, biogenic amines and the factors that influence their creation in other food products are well known. One of the most well-known that influences biogenic amine production is pH, specifically acidity. Two studies [48,49] have shown that when bacteria produce biogenic amines such as tyramine, the bacteria’s ability to survive in acidic environments was greatly improved; in these studies, the non-food LAB *Enterococcus faecalis* was examined, but the results are applicable to food bacteria. In tests conducted at pH’s from 7 to 1, bacterial strains capable of producing tyramine had 50% more surviving cells than those that could not [49]. Chemically, this may happen because biogenic amines are basic compounds, so the decarboxylation of an amino acid not only removes an acidic compound from the microbe’s environment but also produces a base. This base production is seen in a variety of lactic acid bacteria, including the genera commonly used in the brewing of sour beer, like *Lactobacillus* and *Pediococcus* [50]. This implies that in sour beer varieties like gose and lambic may have higher levels of biogenic amines due to the combination of high acidity, lactic acid bacteria, and yeast.

Metabolizable carbon scarcity, primarily of sugars and carbohydrates, can increase biogenic amine production from microbes. Microbes will switch to amino acid digestion when available carbohydrates become scarce, producing biogenic amines and carbon dioxide as waste products. This “starvation” does not appear to affect biogenic amine formation uniformly (Table 11). While little work has been published, specific to beer in this vein, a study focused on sausage fermentation showed that while the concentration of some amines, like spermine and spermidine, are identical regardless of sugar content, the concentration of other amines (tyramine, cadaverine, putrescine, tryptamine, phenethylamine) increased significantly [51]. Compared to sausages with higher concentrations of sugar, tyramine concentration increased by 159%, cadaverine increased 851%, putrescine increased 700%, tryptamine increased 200%, and phenethylamine increased 450%. The reasoning for this change is not well understood, but it is theorized that the lack of carbohydrates causes the microbes to begin decarboxylating amino acids for energy more quickly than if they had abundant carbohydrates [51]. While sausages are a completely different medium, beer does become a low sugar environment post-fermentation, and the only available food source to most bacteria and yeasts are amino acids. There is potential for this phenomenon to become significant in beer that is aged for long periods of time, such as barrel-aged beers.

## 7. Conclusions

The amine component of beer is a complex mixture of amino acids, proteins, and biogenic amines that develops and changes during the brewing process. Proteins are extracted from the barley seed and are then catabolized, solubilized, denatured, and in some cases coagulated. As the brewing process continues, yeasts utilize amino acids to build peptides and as an energy source, causing amino acids to be removed, created, and metabolized, often creating biogenic amines. This amino acid uptake and utilization by yeast and other brewing microbes is poorly understood and necessitates further study.

The chemical complexity of beer paired with previously mentioned unknowns makes analyzing biogenic amine formation and content in beer, challenging. Studies have shown that in other fermented food products, microbial stressors like low pH and nutrient scarcity can increase biogenic amine production, but little has been published on this subject in respect to beer. The rise in *Brettanomyces* fermented beer introduces more uncertainty in the chemical composition of beer. The individual species in this genus produce a diverse range of chemical complexes, but their specific interactions with amino acids and proteins as well as their ability to produce biogenic amines, compounded by the use of a myriad of new amine-containing adjuncts, requires more study.

As microbreweries continue to reinvent the landscape of beer and utilize a wide array of ingredients and techniques, determining the effect these recipes have on the chemical composition of beer becomes ever more essential. Due to their deleterious health effects, determining what biogenic amines are present in finished beer and whether a given style is likely to have high concentrations of these compounds is essential. Identification of specific amino acids present in wort before fermentation, and understanding which amino acids yeast most readily utilize, is a complicated task, but will allow brewers to adjust brewing practices to promote better products while minimizing residual biogenic amines.

## Figures and Tables

**Table 1:** Amino acid composition of barley proteins, adapted from [12].

Nitrogenous Compound	Dry Mass % of Hordein
Aspartic acid	1.2
Glutamic acid	23
Proline	15.3
Glycine	1.7
Alanine	2.2
Valine	3.5
Leucine	4.6
Isoleucine	3.6
Phenylalanine	3.6
Tyrosine	1.6
Tryptophan	0.7
Serine	3.2
Threonine	1.9
Cystine	1.5
Methionine	0.75
Lysine	0.8
Histidine	2.2
Arginine	6
Insoluble humin	0.85
Ammonia	23

**Table 2:** The total amino acid content of hulled and hull-less barley, adapted from [2].

Nitrogenous Compound	Hulled Barley (g/kg, Dry Mass)	Hull-Less Barley (g/kg, Dry Mass)
Protein	13.2	14
Alanine	0.44	0.47
Arginine	0.6	0.64
Aspartic acid	0.71	0.75
Cysteine	0.28	0.31
Glutamic acid	2.98	3.27
Glycine	0.42	0.44
Histidine	0.26	0.28
Isoleucine	0.43	0.46
Leucine	0.79	0.84
Lysine	0.41	0.41
Methionine	0.2	0.28
Phenylalanine	0.68	0.73
Proline	1.32	1.43
Serine	0.54	0.57
Threonine	0.42	0.45
Tryptophan	0.22	0.23
Tyrosine	0.37	0.42
Valine	0.59	0.63

**Table 3:** The soluble protein of commonly used Morex and Harrington barleys, malts, and worts, adapted from [18].

	% of Total Soluble Protein Solubilized by Step		
	Barley	Malt	Wort
Morex			
pH 3.8	17%	56%	27%
Harrington			
pH 3.8	21%	54%	25%
Average			
pH 3.8	19%	55%	26%

**Table 4:** Amino acid profile of five common worts, adapted from [19].

Amino Acids in Wort-Results Expressed as mg Amino Acid/100 mL Wort, 1040 S.G. Gravity					
Amino Acid	Pure Malt	Brewery Wort	Malt + 25% Barley Flour	Malt + 25% Wheat Flour	Proteolytic Malt
Alanine	15.1	9.9	15.5	16.5	39.4
Valine	13.7	9.2	14	14.7	26
Glycine	7.9	5.2	9.1	10.3	19
Iso-leucine	7.5	5.3	9.4	10.7	18.6
Leucine	18.8	10.9	18.3	19.2	39.7
Proline	44.5	38.9	47.9	47.3	73
Threonine	17.2	2.5	11.2	23.9	1.7
Serine	7.4	4.8	7.5	9.3	28.8
Cysteine	neg.	neg.	neg.	neg.	neg.
Methionine	neg.	2.3	4.7	4.9	12.8
Hydroxyproline	neg.	neg.	neg.	neg.	neg.
Phenylalanine	16.2	11.4	15.8	17.3	34.4
Aspartic acid	18.9	17.9	23.3	24.8	29
Glutamic acid	24.8	8	31	40.9	39.6
Tyrosine	8.9	0.8	8.3	8.1	11.5
Ornithine	0.9	neg.	neg.	neg.	13.3
Lysine	10.1	5.6	11.3	8.9	19.2
Tryptophan	trace	neg.	neg.	neg.	neg.
Histidine	trace	0.3	neg.	neg.	0.4
Arginine	15.5	10.8	17.6	13.8	18.3
Cystine	trace	na	neg.	neg.	neg.

Note: na indicates compounds that were not examined, neg indicates compounds that were lost during analysis, trace indicates compounds that gave inconsistent and variable results.

**Table 5:** Amino acids classified by yeast uptake, adapted from [20].

Group A Fast Uptake	Group B Intermediate Uptake	Group C Slow Uptake	Group D Little or No Uptake
Glutamic acid	Valine	Alanine	Proline
Aspartic acid	Histidine	Glycine	
Asparagine	Tryptophan	Ammonia	
Glutamine	Tyrosine		
Serine	Phenylalanine		
Threonine			
Lysine			
Arginine			
Methionine			
Isoleucine			
Leucine			

**Table 6:** Amino acid (AA) profiles and protein concentration in yeast autolysates by high-pressure liquid chromatography, adapted from [32].

	Yeast Species		
		*S. cerevisiae*	*S. stipitis*
Concentration of dry mass (% *w*/*w*)		5.62 ± 0.05	6.40 ± 0.06
Protein Content (g per 100 g of dry mass lysate)		0.54 ± 0.05	0.59 ± 0.01
Concentration of free amino acids (g per 100 g of dry mass lysate)			
Essential AA	Threonine	1.09 ± 0.07	2.44 ± 0.23
	Valine	1.76 ± 0.14	1.91 ± 0.18
	Leucine	2.76 ± 0.15	2.50 ± 0.23
	Isoleucine	1.91 ± 0.15	1.81 ± 0.17
	Methionine	0.78 ± 0.05	0.74 ± 0.06
	Lysine	2.38 ± 0.17	2.35 ± 0.23
	Phenylalanine	1.64 ± 0.12	1.55 ± 0.14
	Histidine	0.86 ± 0.08	0.84 ± 0.07
	Sum	13.18	14.14
Conditionally essential AA	Arginine	5.03 ± 0.35	1.29 ± 0.11
	Cystine	1.39 ± 0.01	1.33 ± 0.08
	Glycine	1.69 ± 0.11	1.18 ± 0.11
	Proline	2.06 ± 0.11	1.20 ± 0.10
	Tyrosine	1.58 ± 0.14	1.55 ± 0.15
	Sum	11.75	6.55
Non-essential AA	Glutamine	2.46 ± 0.20	3.25 ± 0.32
	Serine	11.28 ± 0.72	9.87 ± 0.96
	Asparagine	3.59 ± 0.27	3.69 ± 0.33
	Alanine	2.71 ± 0.25	2.72 ± 0.25 f
	**Sum**	**20.04**	**19.53**
Total sum		**44.97**	**40.22**

**Table 7:** Classification of amino acids by their potential to promote the growth of *Brettanomyces bruxellensis* GDB 248 in aerobiosis or in anaerobiosis [36].

Condition	Preferential	Secondary	Poorly Usable
O_2_	Glutamine	Arginine, Aspartate, Glutamate	Alanine, Asparagine, Cysteine, Glycine, Isoleucine, Lysine, Histidine, Methionine, Phenylalanine, Proline, Serine, Tyrosine, Tryptophan, Valine
N_2_	Aspartate, Glutamate, Glutamic Acid	Alanine, Arginine, Asparagine, Serine, Phenylalanine	Cysteine, Glycine, Isoleucine, Leucine, Lysine, Histidine, Methionine, Proline, Tyrosine, Trptophan, Valine
N_2_ (*Saccharomyces* Yeast)	Aspartate, Aspartic Acid Asparagine, Glutamate, Glutamic Acid, Serine, Threonine, Lysine, Arginine, Methionine, Isoleucine, Leucine	Valine, Histidine, Tryptophan, Tyrosine, Phenylalanine	Alanine, Glycine, Ammonia, Proline

**Table 8:** Results of the determination of amino acids in beer samples (umol/L) [41].

Amino Acid	Non-Alcoholic Pils	Pils	Alt Beer	Non-Alcoholic Wheat Beer	Wheat Beer
Aspartic acid	28.6	---	31.4	187.9	43.9
Glutamic acid	144.2	54.6	43.1	193.8	59.7
Hydroxyproline	6.4	8.5	6.2	4.7	---
Serine	---	9.6	28.1	258.4	39.8
Glycine	208.3	193.2	234.2	316.2	251.3
Histidine	124.1	109.5	63.6	156.7	127.7
Threonine	---	---	20.9	163.3	21.5
Citruline	15.6	8.3	10.7	38	28.1
Alanine	566.1	434.6	242.3	744.4	399.2
Arginine	249.1	223.3	50.7	404.7	50.5
GABA	1315.6	1245.6	1331.5	961.6	890.4
3-Methylhistidine	5.2	---	---	---	---
beta-Aminoisobutryic acid	---	6.3	7	---	---
Proline	3214.7	3402.3	2987.7	1942.7	1959.7
Ethanolamine	147.2	182.3	167.3	116.7	139.4
alpha-Aminobutryic acid	15.6	30.5	---	6.1	---
Tyrosine	227.1	283.2	132.5	298.2	216.5
Valine	295.3	252.8	49.3	513.7	290.2
Methionine	---	0.8	12.2	99.4	20.9
Ornithine	14.7	16.5	20.7	68.4	75.1
Lysine	11.2	10.8	37.7	213	30.2
Isoleucine	50.7	27.9	30.1	250.7	55.8
Leucine	121.1	57.9	53.8	570.3	176.6
Phenylalanine	214.1	203.1	35.1	395.9	213.7
Trytophan	142.9	148.2	55.5	178.4	148.1
Total amino acid content	7117.6	6910	5651.7	8074.3	5238.4

Note: --- indicates missing or unavailable data.

**Table 9:** Analysis of a variety of lager beer from the Czech Republic [42].

Sample No.	Land of Origin			Alcohol (vol%)		Extract of Original Wort (% plato)			Real Extract (wt%)	Real Degree of Fermentation (%)			pH	Color (EBC Units)			Density (g/mL)
1	Czech Republic			4.43		11.76			5.06	58.53			4.73	12.1			1.0116
2	Czech Republic			4.65		11.21			4.11	64.71			4.72	11.4			1.0076
3	Czech Republic			4.76		11.68			4.44	63.44			4.5	27.1			1.0087
4	Czech Republic			4.96		11.47			3.91	67.32			4.62	8.8			1.0075
5	Czech Republic			4.76		11.39			4.13	58.33			4.6	13.5			1.0065
**Concentration (mg/L) of Amino Acids from Same Czech Larger Samples**																	
**Sample No.**	**Asp**	**Ser**	**Glu**	**Gly**	**His**	**Arg**	**Thr**	**Ala**	**Pro**	**Tyr**	**Val**	**Met**	**Lys**	**Ile**	**Leu**	**Phe**	**Total AA**
1	21.3	11.4	17.4	17.5	20.2	32.3	6.3	51.4	241.4	32.2	34.8	5.7	16.1	14.8	25	29.1	576.9
2	9.9	4.6	16.5	11.4	13.9	15.5	2.9	33.1	166.3	26.2	23	3.6	7.3	8.4	16.1	22	380.7
3	9.1	5.7	18.5	7.5	21.1	17.4	3.6	29.1	125.7	37.9	19.4	3.9	9	8.2	13.8	18.4	348.4
4	9.7	6	12.6	14.7	13.8	16	1.5	36.7	200.6	20.4	22.1	2.1	9.9	7.2	9.7	16.5	399.5
5	9.2	4.2	19.4	11.5	30.4	27	3	44.7	232.2	49.5	29.4	4.7	12.8	10.7	19.3	22.9	521.9

**Table 10:** Biogenic amine content of Chinese beer brands (adapted) [45].

Sample No.	Varieties	Beer-Making Areas (Province)	Alcoholic Content (*v*/*v*, %)	Original Gravity (°P)	pH
1	Heineken	Shanghai	≥4.7	11.4	3.98
2	Carlsberg	Guangdong	≥4.0	10.3	4.03
3	Tiger	Shanghai	≥4.7	11.8	4.25
4	Corona Extra	Wuhan	4.6	11.3	4.19
5	Yanjing	Beijing	≥3.6	10	4.13
**Concentration of biogenic amines (ug/mL) (mean ± standard deviation)**					
**Sample No.**	**HIST**	**TRYP**	**TYR**	**PUT**	**SPD**
1	1.20 ± 0.04	ND	6.35 ± 0.06	4.25 ± 0.08	1.00 ± 0.04
2	2.80 ± 0.16	0.45 ± 0.02	5.08 ± 0.12	2.12 ± 0.14	ND
3	0.96 ± 0.03	1.62 ± 0.05	5.65 ± 0.03	2.90 ± 0.06	1.35 ± 0.03
4	1.25 ± 0.03	0.36 ± 0.03	3.47 ± 0.05	4.55 ± 0.05	0.71 ± 0.08
5	4.62 ± 0.13	ND	3.80 ± 0.03	5.73 ± 0.08	1.41 ± 0.14

Note: ND (none detected) indicates none of that compound was detected during analysis.

**Table 11:** Biogenic amine contents of fermented sausages with and without sugar after 20 days of storage (mg/kg) [51].

Biogenic Amine	With Sugar		Without Sugar	
	4 °C	19 °C	4 °C	19 °C
Spermidine	6.5 ± 0.1	5.4 ± 0.1	6.7 ± 0.1	5.6 ± 0.1
Spermine	41.8 ± 0.2	37.2 ± 1.6	41.1 ± 0.6	38.2 ± 1.2
Tyramine	113.9 ± 37.9	114.4 ± 21.1	180.4 ± 4.8	210.2 ± 13.9
Cadaverine	23.6 ± 6.2	31.9 ± 1.6	201.0 ± 11.0	254.1 ± 38.2
Putrescine	4.9 ± 0.3	3.8 ± 1.4	34.5 ± 5.9	42.2 ± 4.4
Tryptamine	0.5 ± 0.3	1.4 ± 0.1	5.6 ± 0.2	10.2 ± 1.2
Phenethylamine	0.4 ± 0.3	0.8 ± 0.2	1.8 ± 0.2	4.5 ± 1.7
